# Selenium phytofortification: enhanced stress resistance and nutraceutical enrichment in horticultural crops

**DOI:** 10.1093/hr/uhaf236

**Published:** 2025-09-03

**Authors:** Yuxi Shangguan, Jin Zhu, Jianhui Ye, Helena Korpelainen, Chunyang Li

**Affiliations:** College of Agriculture and Biotechnology, Zhejiang University, Hangzhou 310058, China; College of Agriculture and Biotechnology, Zhejiang University, Hangzhou 310058, China; College of Agriculture and Biotechnology, Zhejiang University, Hangzhou 310058, China; Department of Agricultural Sciences, Viikki Plant Science Centre, University of Helsinki, PO Box 27, Helsinki FI-00014, Finland; College of Agriculture and Biotechnology, Zhejiang University, Hangzhou 310058, China

## Abstract

As a bridge between human health and plant nutrition, Selenium (Se) phytofortification represents a promising strategy for achieving a safe and effective dietary Se supplementation. Due to chemical similarities, Se absorption, transformation, and storage in crops primarily follow the sulfur metabolic pathway. Se enhances horticultural crop resilience against abiotic and biotic stresses by: (i) boosting antioxidant capacity, (ii) inducing hormonal cascades, (iii) promoting the accumulation of key metabolites (e.g. amino acids, flavonoids), (iv) strengthening cellular functions, and (v) harnessing plant–microbiome interactions. In horticultural crops, most Se exists in organic forms, such as selenoamino acids, selenoproteins, selenium-polysaccharides, and selenium-polyphenols, which contribute to unique quality traits. Additionally, Se regulates the synthesis of core nutrients, including amino acids, flavonoids, phenolic compounds, soluble sugars, mineral elements, alkaloids, and volatile compounds. It also extends postharvest shelf life by delaying senescence and deterioration. Current phytofortification strategies focus on enhancing bioavailable Se in edible parts through agronomic interventions and plant breeding. Artificial Se fertilization is the most common agronomic approach, classified by the application method (soil fertilization, foliar spraying, hydroponic supplementation, and seed soaking) and fertilizer type (inorganic, organic, nano-Se, and biosynthesized fertilizers). Optimizing plant species, fertilization methods, dosage, timing, and elemental synergies maximize phytofortification efficiency.

## Selenium phytofortification: bridging human health and plant nutrition

Selenium (Se) is an essential trace element vital for human health [1]. Due to a highly uneven global soil Se distribution, ~1 billion people suffer from Se imbalance [2]. Se deficiency (<40 μg/d) can lead to infertility, Keshan disease, Kashin–Beck disease, hypothyroidism, and cognitive impairment, afflicting >15% of the population [3, 4]. Conversely, excess Se intake (>400 μg/d) causes vomiting, pain, and nausea [4]. Hence, developing strategies for safe Se supplementation in humans has become a focus research area. Dietary organic Se demonstrates lower toxicity than inorganic Se and is more readily absorbed and utilized by the human body [5]. Through Se phytofortification, horticultural crops not only accumulate higher Se contents but also often exhibit an enhanced quality and flavor profiles, making them optimal dietary Se sources for humans [6]. Se phytofortification is a biofortification technique that enhances the Se content in edible parts of horticultural crops through agronomic or breeding strategies, enabling safe and effective dietary Se supplementation.

In general, the concentration and chemical form of Se in plants are determined by their inherent accumulation capacity [7]. Based on their accumulation capacity, plants are classified as nonaccumulators (<100 μg/g dry weight (DW), low suitability for phytofortification), Se-accumulators (100–1000 μg/g DW, primary targets for Se phytofortification), and Se-hyperaccumulators (>1000 μg/g DW, ideal for phytofortification but rare in crops) [7, 8]. Most plants are nonaccumulators. Species like cardamom, broccoli, garlic, onion, *Stipa*, *Astragalus*, *Atriplex*, *Melilotus*, mustard, tomato, radish, potato, strawberry, tea, and nuts exhibit strong Se accumulation [2, 9–13]. Although Se is non-essential for plants [14], phytofortification shows positive effects [15] like enhancing nutritional contents, improving key sensory attributes (e.g. sweetness, aroma, color), and boosting antioxidant capacities to mitigate abiotic/biotic stresses. This review synthesizes mechanisms of Se phytofortification in horticultural crops, its roles in quality enhancement and stress resilience, and proposes optimization strategies for agricultural applications.

## Dynamic network of Se metabolism: uptake, transport, transformation, and storage

**Figure 1:**
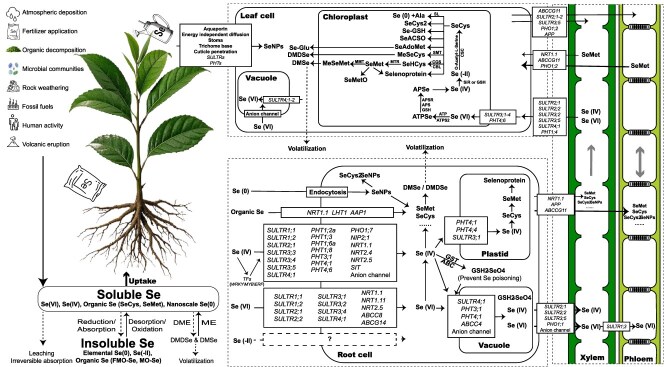
A framework of the transformation of Se in nature and its absorption, transport, and metabolism in plants. Se (VI): selenate, Se (IV): selenite, Se (-II): selenide, Se (0): elemental Se, SeCys: selenocysteine, SeMet: selenomethionine, SeCys2: selenocystine, SeNPs: Se nanoparticles, SeCys2SeNPs: selenocystine-functionalized Se nanoparticles, SeMetO: selenomethionine S-oxide, MeSeMet: methylselenomethionine, SeHCys: selenohomocysteine, ME: methylation, DME: demethylation, DMSe: dimethyl selenide, DMDSe: dimethyl diselenide, SULTR: sulfate transporter, PHT: phosphate transporter, SIT: silicon influx transporter; LHT: lysine/histidine transporter, NRT: nitrate transporter, NIP: nodulin 26-like intrinsic protein, AAP: amino acid permease; ABC: ATP-binding cassette transporters, GST: glutathione S-transferase, GSH: glutathione, APS: adenosine phosphosulfate, APSR: adenosine phosphosulfate reductase, APSe: adenosine phosphoselenate, SL: selenocysteine lyase, Ala: alanine, Se-GSH: selenodiglutathione, SeACSO: seleno-acetylcysteine sulfoxide, MeSeCys: selenomethylselenocysteine, Se-Glu: selenoglutathione, MMT: methylselenol methyltransferase, MTR: 5-methyltetrahydrofolate-homocysteine methyltransferase, SMT: selenocysteine methyltransferase, CGS: cystathionine *γ*-synthase, CBL: cystathionine *β*-lyase, CGS: cystathionine *γ*-synthase, CBL: cystathionine *β*-lyase, SiR: sulfite reductase, CSC: cysteine synthase complex, containing serine acetyltransferase and *O*-acetylserine (thiol) lyase, FMO-Se: Se absorbed by soil metals like Fe and Mn, OM-Se: Se absorbed by soil organic matter.

Se occurrence forms a rank by its bioavailability: selenate (VI) > organic Se > selenite (IV) > elemental Se (0) > selenides (-II) [16, 17]. Due to a chemical similarity with sulfur (S), Se shares metabolic pathways with S [2]. Figure 1 illustrates the pathways of Se uptake, transport, transformation, and storage in horticultural crops.

The absorption stage includes underground absorption and aboveground absorption. The absorption of underground parts is dominated by plant roots and relies on multiple transporters. So far, no specialized Se transporter protein has been identified in horticultural crops [18]. As shown in Table 1, selenate mainly relies on sulfate transporters, selenite on phosphate transporters, organic Se on amino acid and nitrate transporters, as well as on ABC transport proteins, and nano-Se mainly relies on direct penetration (pore size between 5 and 20 nm), passive diffusion, or endocytosis entering the root system [17, 29, 30]. This process is influenced by soil properties (pH, Eh, microbiota) and plant factors (species, growth stage) [18, 19, 31, 32]. The aboveground absorption organs mainly involve leaves and fruits, which primarily rely on stomatal infiltration (10- to 100-μm apertures) and cuticular permeation, with efficiency modulated by surface waxes and trichomes [33–36].

**Table 1:** Se transporters in horticultural crops

Transporter	Key regulation	Response Se form	Tissue localization	References
*SULTR1;1*	Root uptake at low concentrations	Se^6+^, Se^4+^	Root epidermal membrane	[19]
*SULTR1;2*	Root uptake at low concentrations; Intertissue loading; Root-to-aerial tissue transport	Se^6+^, Se^4+^	Thin-walled cell membranes on the surface and xylem of roots	[18]
*SULTR1;3*	The flux of Se/S through xylem and phloem regulation			[20]
*SULTR2;1*	Root uptake at high concentrations; Intertissue loading; Root-to-aerial tissue transport; The flux of Se/S through xylem and phloem regulation	Se^6+^, Se^4+^	Leaf/root xylem parenchyma cells and root pericycle cells	[17, 20]
*SULTR2;2*	Xylem thin-walled tissue, bundle sheath cells, and chloroplastic tissue redistribution; The flux of Se/S through xylem and phloem regulation	Se^6+^	Xylem parenchyma cells, columnar sheath, or periductal cytoplasmic membrane	[17, 20]
*SULTR3;1*	Translocation from cytoplasm to plastid in leaf cells; The flux of Se/S through xylem and phloem regulation	Se^6+^	Chloroplast membranes and cell membranes	[20, 21]
*SULTR3;2*	Storage/release in vesicles; Redistribution/storage in foliage and phloem	Se^6+^	Phloem or tonoplast	[19]
*SULTR3;3*	Root uptake at high concentrations	Se^4+^	Cortical cell membrane or tonoplast	[19]
*SULTR3;4*	Transcellular transport from the cortex to the midpostal sheaths	Se^6+^, Se^4+^	Root cortex and pericycle cell membranes	[22]
*SULTR3;5*	Se^4^ + −specific signaling pathway; Phloem to chloroplast transport	Se^6+^, Se^4+^	Phloem or phloem cell membrane	[19]
*SULTR4;1*	Root uptake, translocation; Vesicle redistribution	Se^6+^, Se^4+^	Tonoplast	[19]
*PHT1;2a*	Root uptake	Se^4+^	Epidermal cell plasma membrane, endoplasmic reticulum	[23]
*PHT1;3*	Root uptake	Se^4+^	Root/stem cell plasma membrane, vesicles	[22]
*PHT1;4*	Leaf uptake	Se^6+^	Leaves	[22]
*PHT1;6a*	Root uptake	Se^4+^	Root epidermal cell plasma membrane	[22]
*PHT1;8*	Root uptake	Se^4+^	Root epidermal cell plasma membrane	[23]
*PHT3;1*	Root uptake at low concentrations; Storage/redistribution in mitochondria or vesicles	Se^4+^	Mitochondria, tonoplast, chloroplast, prexisome	[22]
*PHT4;1*	Root uptake at high concentrations; Golgi/cytoplasmic transport	Se^4+^	Plasma membrane, vesicles	[18]
*PHT4;6*	Root uptake at high concentrations;	Se^4+^	Plasma membrane, chloroplast, stem	[18]
*PHO1;1*	Root-to-aerial tissue transport	Se^4+^	Plasma membrane, Golgi apparatus	[24]
*PHO1;2*	Vesicle storage; Translocation of SeMeCys in the aboveground tissue	Se^4+^, organic Se	Nucleus, chloroplast	[24]
*PHO1;7*	Transmembrane signaling	Se^4+^	Root	[24]
*NIP2;1*	Passive transmembrane transport	Se^4+^	Root epidermal cell plasma membrane	[25]
*SIT*	Root uptake	Se^4+^	Root epidermal cell plasma membrane	[25]
*NRT1.1*	Root uptake; Root-to-aerial tissue transport of SeMet	Se^6+^, Se^4+^, organic Se	Plasma membrane	[26]
*NRT1.11*	Uptake and distribution	Se^6+^		[27]
*NRT2.4*	Uptake and distribution	Se^4+^		[27]
*NRT2.5*	Uptake and distribution	Se^6+^, Se^4+^		[27]
*ABCC8*	Uptake and tolerance	Se^6+^		[27]
*ABCG14*	Uptake and tolerance	Se^6+^		[27]
*ABCCG11*	Loading of organic Se in phloem; Redistribution from mature leaves to new shoots; Root-to-aerial tissue transport; Synergistic regulation with the PHO family	Se^4+^, organic Se	Leaves, phloem cell membrane	[27]
*ABCC4*	Maintenance of dynamic equilibrium; Toxicity tolerance	Se^6+^, Se^4+^, organic Se	Tonoplast	[27]
*LHT1*	Uptake	Organic Se	Phloem cell membrane	[28]
*AAP1*	Uptake and transport of SeCys and SeMet	Organic Se	Plasma membrane	[28]

SULTR: Sulfate Transporter; PHT: Phosphate Transporter; SIT: Silicon Influx Transporter; LHT: Lysine/Histidine Transporter; NRT: Nitrate Transporter; NIP: Nodulin 26-like Intrinsic Proteins; AAP: Amino Acid Permease; ABC: ATP-binding Cassette Transporters.

The distribution of Se in plants follows the pattern of mature tissues>young tissues, phloem>xylem, and underground parts>aboveground parts, which is determined by the transport mechanism of Se. The transport of Se in plants involves Se transporters (SeT), Se amino acid transporters (SAT), and selenoproteins. They synergistically mediate absorption, transmembrane transport, and dynamic distribution [23, 25]. During underground absorption, selenite undergoes rapid conversion to organic Se in roots, with only a small portion transported to shoots [23, 27, 28, 37]. Selenate, however, displays superior mobility via symplastic pathways to leaves for bioconversion, followed by phloem-mediated redistribution of organic Se [17, 21, 27, 38]. Nano-Se oxidizes to selenite in roots/leaves before apoplastic/symplastic transport to shoots [29, 30]. During aboveground absorption, inorganic Se is predominantly converted to organic forms, primarily existing as soluble proteins. A portion can translocate to new buds and leaves through phloem conduits, but there is no downward mobility to roots [29, 33].

Transformation involves inorganic activation and organic conversion. Selenate (SeO_4_^2−^) is activated via ATP sulfurylase (ATPS2) to form 5′-phosphoselenoadenosine (ATPSe). Subsequently, ATPSe undergoes conversion to adenosine 5′-phosphoselenate (APSe) through the enzymatic actions of APS reductase (APSR) and APS kinase with reduced glutathione (GSH) as an electron donor, ultimately yielding selenite (SeO_3_^2−^) [1, 21, 27, 39]. The reduction of SeO_3_^2−^ to Se^2−^ occurs via two pathways: a direct enzymatic reduction by sulfite reductase (SiR) in chloroplasts or a reduction through a GSH-mediated GSSeSG→GSSeH cascade. Se^2−^ combines with *O*-acetylserine via the cysteine synthase complex to form selenocysteine (SeCys) [40, 41]. It has been speculated that SeCys are transformed through eight metabolic pathways during the mechanical transformation stage [28, 39, 42, 43]: (i) Decomposition to Se(0) and alanine by selenocysteine lyase (SL). (ii) Oxidative coupling to selenocystine (SeCys₂). (iii) Synthesis of selenoglutathione (Se-GSH) via *γ*-glutamylcysteine synthetase and GSH synthetase. (iv) Conversion to selenohomocysteine (SeHCys) by cystathionine-*γ*-synthase (CGS) and cystathionine-*β*-lyase (CBL), followed by SeHCys→selenomethionine (SeMet) transformation via methionine synthase (MTR). Partial SeMet integrates into selenoproteins or is methylated by methionine methyltransferase (MMT) to volatile dimethylselenide (DMSe). (v) Formation of Se-allyl-*L*-cysteine sulfoxide (SeACSO) under stress. (vi) Synthesis of selenoadenosylmethionine (Se-AdoMet) and selenoglucosinolates. (vii) Methylation to methylselenocysteine (MeSeCys) via selenocysteine methyltransferase (SMT), yielding volatile dimethyl diselenide (DMDSe). (viii) Nonspecific incorporation into proteins, which may disrupt molecular conformation, impairs functional integrity, and ultimately triggers phytotoxicity. Methylation and volatilization are key mechanisms for Se tolerance. Metabolites, such as SeCys₂, Se-GSH, and selenoglucosinolates, are likely to contribute to antioxidant defense, detoxification, and Se storage/transport [21].

## Se-induced multidimensional stress resistance

**Figure 2:**
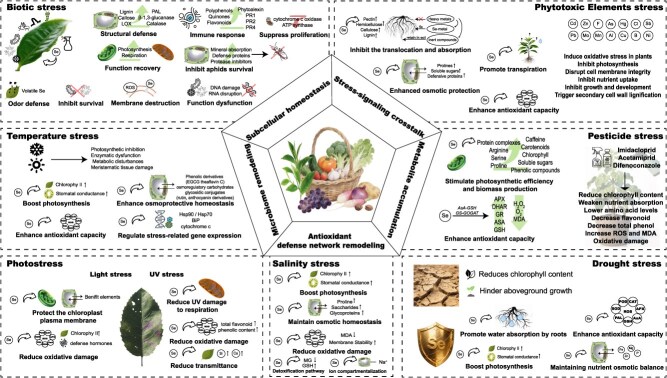
Se-induced multidimensional stress resistance mechanisms. LOX: lipoxygenase, PR: pathogenesis-related protein, PAL: phenylalanine ammonia-lyase, ROS: reactive oxygen species, CAT: catalase, APX: ascorbate peroxidase, AsA: ascorbic acid, GSH: glutathione, EGCG: epigallocatechin gallate, Hsp: heat shock protein, Bip: binding immunoglobulin protein, Si: silicon, MG: magnesium, GSH: glutathione, Ca: calcium, Cd: cadmium, Zn: zinc, F: fluorine, As: arsenic, Hg: mercury, Cl: chlorine, Sb: antimony, Pb: lead, Mo: molybdenum, Mn: manganese, Al: aluminum, Cu: copper, B: boron, Ni: nickel, GS-GOGAT: glutamine synthetase-glutamate synthase cycle, AsA-GSH: ascorbate -glutathione cycle, H_2_O_2_: hydrogen peroxide, O_2_^−^: superoxide anion radical, MDA: malondialdehyde, DHAR: dehydroascorbate reductase, GR: glutathione reductase.

Se is essential for plant growth, development, and signaling, and it functions as a plant growth regulator [1, 44]. It stimulates plant metabolism, participates in the defense compound synthesis, and mitigates environmental stresses [14, 45, 46]. As shown in Fig. 2, Se enhances resistance in most horticultural crops against multiple stresses, including moisture, temperature, photo, salinity, phytotoxic, and biotic stresses [47].

As shown in Table 2, the mechanisms can be summarized as the following five core points: First, Se enhances plants’ antioxidant capacity. On the one hand, it elevates non-enzymatic antioxidants (e.g. Se-containing proteins for reactive oxygen species (ROS) scavenging, vitamin C, vitamin E, anthocyanins) [13, 35, 43, 61]. On the other hand, Se synergistically regulates genes encoding enzymatic antioxidants, including Cu-Zn-SOD and glutathione peroxidase (GPX), lipoxygenase (LOX), catalase (CAT), peroxidase (POD), superoxide dismutase (SOD), and ascorbate peroxidase (APX) in plants, thus enhancing their activity [13, 48, 53, 55, 61, 62]. This dual action reduces oxidative markers (ROS, malondialdehyde (MDA), tocopherols) and protects membrane integrity [2, 51].

**Table 2:** Effects of selenium on stress resistance of horticultural crops

Crop species	Treatment	Stress	Influence	References
Okra (*Abelmoschus esculentus* L.)	Foliar application	Drought	Reducing chlorophyll and carotenoid content; increasing anthocyanin, proline, peroxidase, ascorbate peroxidase, and catalase activities; significantly alleviating biochemical interference caused by drought stress	[48]
Garden strawberry (*Fragaria × ananassa* Duch.)	Foliar application 0, 2, 4 ppm sodium selenate	Drought	Enhancing plant morphology and relative water content; increasing fresh fruit yield, total phenolic and flavonoid content; alleviating the negative effects of stress	[45]
Onion (*Allium cepa* L.)	Foliar application 0, 15, 30, 45, 60 g/ha Se	Drought	Increasing plant height and dry matter accumulation to increase bulb yield	[49]
Quinoa (*Chenopodium quinoa* Willd.)	Seed priming 0, 3, 6, 9 mg/l Se	Drought	Maintaining plant water status; improving main spike length, main spike weight, 1000-grain weight, stomatal conductance, and photosynthetic rate; increasing total phenolic and chlorophyll contents; enhancing grain yield; elevating P, K, and protein contents for grain quality; alleviating adverse effects of drought.	[50]
Chili pepper (*Capsicum annuum* L.)	Foliar application	Chromium stress	Improving plant growth, biomass, and antioxidant capacity; reducing the bioavailability of Cr; limiting lipid peroxidation and membrane damage	[51]
Tomato (*Solanum lycopersicum* L.)	Foliar application 15–60 μM/l SeNPs	Cadmium stress	Mediating the synthesis of Se/S related genes and key enzyme activities; promoting the accumulation of thiol compounds; inducing the synthesis of plant chelating agents; enhancing the storage of Cd in roots; inhibiting the transport of Cd to shoots; reducing the plant toxicity of Cd	[20]
*Agaricus blazei* Murill	0, 10, 20, 50 mg/kg SeNPs in substrates	Cadmium stress	Restricting Cd translocation to fruiting body; reducing oxidative damage; alter metabolism	[13]
*Artemisia argyi*	Soil application 0, 0,5, 1, 2 mg/kg Se	Cadmium stress	Increasing seedling biomass, permeable substances; enhancing antioxidant capacity; reducing MDA content; decreasing Cd accumulation in cell walls and soluble components	[52]
Cucumber (*Cucumis sativus* L.)	Foliar application 25 mg/l nano-Se	Combined salinity and heat stress	Improving the growth parameters (plant height and leaf area); increasing the photosynthetic rate; increasing the concentrations of N, P, K, Mg, Se, and Si in the leaves	[53]
Tea (*Camellia sinensis* (L.) Kuntze)	Foliar application 0, 20 mg/ml Na_2_SeO_3_	Low temperature	Increasing the net photosynthetic rate of leaves; decreasing the content of hydrogen peroxide and MDA; increasing the content of proline, polyphenols, carbohydrates, and amino acids; increasing the activities of SOD, APX, POD, and CAT	[54]
Tomato (*Solanum lycopersicum* L.)	Foliar application 5 μM Se	Alkaline stress	Increasing the aboveground biomass, leaf photosynthetic pigments, relative water content of leaves, proline content, and antioxidant enzyme activity; reducing the content of Na^+^, Na^+^/K^+^ ratio, hydrogen peroxide, MDA, and MSI	[55]
*Cucumis sativus* L.	Foliar application 0, 3, 6, 12 mg/l Se-enriched ionic fertilizer	Powdery mildew	Increasing the activities of superoxide dismutase and peroxidase; inhibiting the germination of powdery mildew pathogen conidia and mycelium formation; 6 mg/l Se efficacy achieving comparable to powdery mildew drug treatment	[56]
Garden strawberry (*Fragaria × ananassa* Duch.)	1 mg/l Na_2_SeO_4_ in nutrient solution, and 200 μM NaHS by foliar application	Salt stress	Significantly inhibiting free radicals; increasing anthocyanin, vitamin C content, and antioxidant enzyme activity; enhancing flavor, and increasing fruit yield and size	[13]
*Brassica rapa* L.	Used Se (75, 100, 125 μmol/l) as the seed priming agent	Salt stress	Upregulating the expression levels of antioxidant genes such as CAT, POD, SOD, to eliminate ROS; increasing seed germination rate, photosynthetic content, and seedling biomass	[57]
*Carthamus tinctorius* L.	Soil application 0, 0.01, 0.02 g/kg selenate	Salt stress	Reducing the negative effects of salt stress; enhancing POX and PPO activity in leaves and roots; promoting the synthesis of secondary metabolites such as phenols, flavonoids, and anthocyanins	[58]
*Dianthus barbatus* L.	Foliar application 0, 5, 10, 15 μM	Salt stress	Increasing phenolic and flavonoid contents; enhancing antioxidant capacity; improving salinity tolerance	[59]
*Aloysia citrodora* Paláu	Foliar application 10 μM nano-Se and 10 μM Se	Salt stress	Promoting the accumulation of osmolytes (proline, soluble sugars, and total proteins); enhancing antioxidant activity; reducing electrolyte leakage, MDA, and hydrogen peroxide (H_2_O_2_) accumulation in leaves; boosting biosynthesis of secondary metabolites (essential oils, total phenolics, and flavonoids); decreasing Na accumulation in roots and shoots under stress	[60]

Second, Se mediates phytohormone signaling and metabolite synthesis, thereby mitigating toxin. Se modulates phytohormone-signaling pathways. It induces expression of stress-responsive genes (e.g. ERF, MYB, and WRKY families in tea [22]; IAA, GH3, and SAUR in cucumber [62]). Moreover, Se orchestrates hormonal cascades involving jasmonic acid (JA), methyl jasmonate (MeJA), salicylic acid (SA), abscisic acid (ABA), ethylene, and cytokinins [1, 20]. This reconfigures plants’ metabolism and defense responses to improve stress tolerance [61]. On the other hand, Se enhances the synthesis of osmolytes like soluble sugars, proline, phenolics, flavonoids, glycine betaine, and trehalose to maintain osmotic balance under stress [48, 53, 55, 58, 63]. Moreover, Se inhibits toxic metal uptake and translocation by regulating transporter genes, synthesizing phytochelatins, forming inert Se-metal complexes, altering the composition of root cell walls and subcellular distribution of roots [64]. It also improves nutrient acquisition, supporting growth under adversity. Besides, it reduces oxidation products of tea polyphenols (e.g. theaflavin), bolstering antioxidant capacity [65].

Third, Se enhances cellular functions. Se can significantly enhance the growth characteristics of plants [45, 55, 66], such as promoting the distribution of carbon (C) assimilates to roots to increase the length of main roots and the number of lateral roots, thereby promoting the absorption of nutrients. Research on crops such as tea [61], tomatoes [55], strawberries [13], cucumbers [53], and roses [63] has found that Se can increase the content of photosynthetic pigments and enhance photosynthesis [49, 57]. When under stress, Se not only prevents the degradation of chlorophyll, but also synthesizes Fe–Se complexes to provide sufficient substrates for the high excitation energy of electron levels to maintain tissue, reduce ROS production, and alleviate the adverse effects of stress on the photosystem II and photochemical efficiency [13, 35]. Se plays a critical role in maintaining normal mitochondrial number and cristae density in plants under stress conditions. Moreover, Se participates in the synthesis of respiratory chain coenzymes, enhances the activity of the *α*-ketoglutarate oxidase system, and increases the mitochondrial respiratory intensity, thereby exerting essential biological functions in the tricarboxylic acid (TCA) cycle and electron transport chain [67]. Se regulates stomatal aperture and closure [45], thereby maintaining the tissue water content and enhancing water use efficiency under drought, salinity, and metal stress [55]. This mechanism protects the cellular structure and function. Consequently, Se does not adversely affect biomass [53]. For certain crops, appropriate Se doses promote nutritional growth, accelerate biomass accumulation, and shorten germination time [2, 34, 61, 68, 69].

Microbe–plant interactions represent a key mechanism for Se-mediated stress resistance enhancement. Endophytic symbionts within plant tissues promote Se accumulation and significantly regulate proline levels and stress-marker enzymes, thereby strengthening plants’ stress defense [12]. Concurrently, Se enriches functional endophytes associated with nitrogen (N) fixation, ammonia assimilation, toxic substance degradation, environmental adaptation, energy metabolism, and DNA repair, fostering plant growth and stress resilience [12, 70, 71]. Additionally, plant growth-promoting rhizobacteria (PGPR) enhance root Se uptake by increasing rhizosphere bioavailability and upregulating transporter gene expression [16, 17, 72], while improving tolerance to high soil Se levels [12]. Se also remodels the rhizosphere microbiome by altering root exudates and the rhizospheric environment [73], recruiting beneficial microbes or suppressing pathogens. This stabilizes ecosystems and reduces disease susceptibility [26, 34, 74]. For example, Se can alter the metabolic product distribution of citrus root exudates, increase the abundance of key substances, reshape the microbial community, and improve soil phosphorus utilization efficiency [73]. Furthermore, Se exhibits antimicrobial (*Sclerotinia sclerotiorum*), antiviral, and nematicidal properties, synergizing with fungicides to protect crops against biotic stresses [56, 75]. However, the effect is mainly inhibitory rather than lethal.

## Selenium-driven enrichment of nutritional products

In Se-enriched crops, the majority of Se exists in organic forms. Organic Se primarily resides in free selenoproteins and amino acids, with minor fractions bound to pigments, polyphenols, pectin, and nucleic acids [2, 61]. Common seleno amino acids include *γ*-glutamyl-selenium-methylselenocysteine, *γ*-glutamyl-selenomethionine, SeMet, selenium-methyl-selenocysteine (SeMSC), SeCys, and MeSeCys [2]. These compounds form selenoproteins by nonspecifically replacing Cys and Met in proteins, thus disrupting protein folding and function [76]. Currently, no specific SeCys insertion mechanism has been identified in higher plants [36]. Selenoproteins mediate Se sequestration, stress resistance, secondary metabolism, photosynthesis, antioxidant responses, and S/Se metabolic pathways, including glutathione peroxidase (GSH-Px), CAT, ATP synthase subunit a, and thioredoxin reductase (TRx) [77]. Selenopolysaccharides (Se-TPS) consist of a variety of selenoproteins and polysaccharides linked by covalent bonds of -OH, -NH2, -COOH, sugar chains, and peptide chains to form Se-H, C-O-Se, O-Se-O, Se-O, and Se=O bonds. These unique covalent bonds endow Se-TPS contributes to cell wall and have a higher antioxidant activity, which can enhance the resistance of tea to stress [78, 79]. Selenoflavonoids account for ~1% of total Se. The nucleophilic hydroxyl group at the C-3-OH position of the flavone benzopyran ring reacts with HSeO_3_^−^, forming Se-O-C and Se=O bonds that replace the hydroxyl group. This structural modification disrupts molecular planarity, reduces intermolecular attraction, and improves cellular uptake, thereby amplifying antioxidant and pharmacological effects [40, 41].

**Figure 3:**
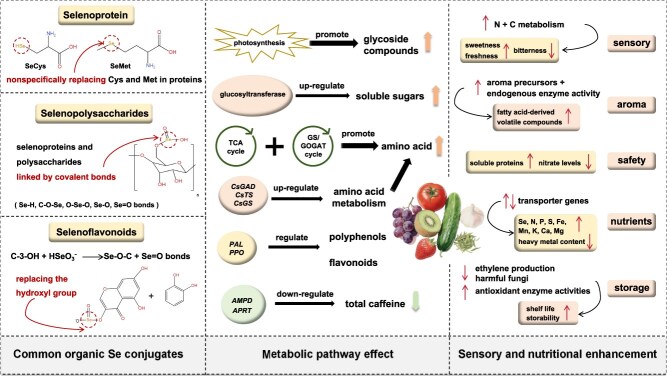
A quality regulation network of selenium in horticultural crops. SeCys: selenomethionine, SeMet: selenomethionine, GS/GOGAT: glutamine synthetase/glutamate synthase, PAL: phenylalanine ammonia-lyase, N: nitrogen C: carbon. The majority Se exists as organic forms, primarily selenoamino acids and Se-complexes (Se-polysaccharides, Se-flavonoids). Se modulates N, C, and secondary metabolism to reconfigure quality-component profiles, thereby enhancing sensory attributes, optimizing mineral nutrients and extending shelf life and storability.

As Fig. 3 shows, Se regulates the composition and content of amino acids, flavonoids, phenolic compounds, soluble sugars, mineral nutrients, alkaloids, and volatile substances in horticultural crops. These changes enhance sensory attributes and nutritional value [15, 45]. For instance, Se can significantly enhance the sweetness, freshness, and aroma of tea, while reducing bitterness [11, 18, 45, 61, 68]. The reason is that the quality effect of Se on horticultural crops is potentially associated with N and C metabolism, showing concentration dependence and variety specificity. For example, in tea, a low Se concentration mainly affects carbohydrate and flavonoid metabolism, while a high Se concentration mainly regulates amino acid metabolism [18, 37, 67]. The concentration and composition of carbohydrates are important components of the flavor quality of horticultural crops. The improvement effect of Se on photosynthesis also contributes to the synthesis and accumulation of glycoside compounds [13]. Se upregulates glucosyltransferases, significantly increasing soluble sugars (glucose, fructose, sucrose) in leaves or fruits [15, 34, 61, 80]. Proteins and amino acids are important N-containing compounds. Se regulates the glutamine synthetase/glutamate synthase (GS/GOGAT) cycle, TCA cycle, and amino acid metabolism. It upregulates the genes of key enzymes of amino acid biosynthesis, including glutamate decarboxylase (CsGAD), theanine synthase (CsTS), glutamine synthetase (CsGS), and pathways for glycine, serine, and threonine metabolism. Consequently, Se modulates the biosynthesis of multiple essential plant amino acids [18, 34, 54, 62, 81, 82]. It has been found that Se can also increase the concentration of soluble proteins in crops and reduce nitrate levels, thereby improving the safety quality of crops [80, 83].

In horticultural crops, secondary metabolites are closely associated with their health benefits. Phenolic compounds and flavonoids represent the two most vital classes of secondary metabolites in plants. Ubiquitous across all plant species, they govern fruit flavor and coloration [84]. Se modulates the expression of key enzyme genes in polyphenol and flavonoid biosynthesis pathways, thereby influencing the polymerization, isomerization, and metabolic direction of polyphenolic compounds. The key enzyme genes include ammonia-lyase (PAL), 4-coumarate-CoA ligase (4CL), flavonol synthase (FLS), polyphenol oxidase (PPO), and so on. Consequently, Se alters the content and composition of polyphenols and flavonoids [18, 29, 34, 40, 43, 60, 81, 85]. For alkaloids, Se may downregulate key genes in the caffeine biosynthesis pathway, such as AMP deaminase (AMPD) and adenine phosphoribosyltransferase (APRT), thereby reducing the total caffeine content in crops [29, 40, 82]. Se regulates the synthesis of aroma precursors and endogenous enzyme activities, triggering the formation of fatty acid-derived volatile compounds and altering volatile component profiles, thereby optimizing crop aroma [61, 67]. The interaction between Se and plant mineral nutrients is dose-dependent [38]. Appropriate Se doses optimize nutrient homeostasis and enhance crop quality by influencing plants’ physiological metabolism and regulating transporter genes (e.g. CsIRTs, CsZIPs, CsBOTs) [45, 86]. For instance, Se elevates the contents of Se, N, P, S, Fe, Mn, K, Ca, and Mg in crops, such as grapes [87], strawberries [45], tomatoes [38, 55], and cucumbers [53]. Overall, Se increases essential macro/micronutrient accumulation while reducing heavy metal content, although specific elemental responses differ among plant species [15, 72, 88]. Additionally, Se phytofortification enhances the shelf life and storability of horticultural crops, exerting beneficial effects on postharvest preservation in certain species. For example, Se can extend the vase life of cut roses [63]. These effects are attributed to Se reducing ethylene production, elevating antioxidant enzyme activities in fruits, and inhibiting the germination and proliferation of harmful fungi [2, 84].

## Application strategy for Se phytofortification

Se phytofortification relies on plant breeding and agronomic interventions [2]. Artificial Se fertilization is a common agronomic approach, current methods including soil fertilization, foliar application, hydroponic supply, and seed soaking [2, 14, 57, 69]. Soil fertilization offers operational simplicity and persistent Se enrichment but risks Se fixation, leaching losses, low bioavailability, and environmental contamination [16]. Foliar application demonstrates a higher phytofortification efficiency than soil fertilization [15], characterized by rapidity, safety, and cost-effectiveness [33, 36]. It is widely adopted for horticultural crop phytofortification. However, foliar spraying is susceptible to adverse weather conditions (e.g. strong winds, heavy rain, frost) and may leave Se residues [82]. Comparatively, a hydroponic Se supply provides operational simplicity, reproducibility, and high absorption efficiency, achieving effective phytofortification in vegetables, such as sweet basil, lettuce, tomato, spinach, and endive [2, 14]. Seed soaking with Se solutions is primarily applied to cereals, with limited research on its efficacy in horticultural crops.

The efficacy of Se phytofortification exhibits concentration dependence and cultivar specificity, influenced by target crops, Se fertilizer forms, concentrations, application methods, timing, and environmental conditions. For example, spraying Se during the peak vegetative growth stage of crops can achieve the optimal effect [89]. Current agricultural Se fertilizers are categorized into four types: inorganic Se (rapid absorption, low cost, and persistence, but unstable efficacy, narrow safety threshold, and potential soil compaction), organic Se (high bioavailability, enhanced safety, and slow-release properties, yet costly and slow-acting), nano-Se (lower toxicity, stability, high bioactivity, and environmental friendliness), biosynthetic Se (high biological efficacy, safety, and bioavailability) [26, 34, 39, 90, 91]. These fertilizer types differ significantly in mobility, adsorption traits, and application outcomes, necessitating comprehensive evaluation of soil fixation, bioavailability, and environmental risks.

Se exerts biphasic concentration-dependent effects on plants [82]. The safe dosage range varies for different types of Se fertilizers and crops. Based on existing knowledge, the appropriate dosage range for most vegetables is 2–15 mg/l [56]. Low-dose Se acts as an antioxidant, while high concentrations promote oxidation [14]. Excess Se induces oxidative damage, photosynthetic inhibition, and metabolic disorders, leading to wilting, stunted growth, yield loss, or plant death [21, 42, 45, 61, 92]. Plants detoxify Se via *β*-alanine, taurine, hypotaurine, and S assimilation pathways, where GSH scavenges ROS and glutathione S-transferase (GST) mediated metabolism plays key roles, particularly for toxic selenite [22, 27, 39, 65, 92]. An overapplication risks ecosystem disruption and food chain contamination, demanding strict dosage control. The Se accumulation capacity varies by crop cultivar [49], season [29], and growth stage [10, 46]. For example, tea cultivars Zhongcha 108, Echa 10, Fuding Dabai, and Longjing 43 have been found to accumulate more Se, whereas Jin Guanyin, Tie Guanyin, Wuniuzao, Jiuhan, and Liuye accumulate less Se [18, 26, 32]. Breeding strategies (conventional or transgenic) to enhance Se accumulation in edible parts (e.g. leaves/fruits) hold significant potential for phytofortification.

Se interacts synergistically with N, phosphorus (P), and S in plant metabolism [26, 93]. N promotes Se transport and bioavailability [21, 94]. Exogenous N (NH_4_^+^/NO_3_^−^) elevates Se and amino acid contents in crops [26]. Se regulates N-metabolism enzymes (e.g. CsTS1, AMT, GS, GOGAT) and microbiome-driven N cycling, optimizing N-use efficiency and alleviating excessive N stress [26, 68, 94]. Moderate P enhances the Se uptake, photosynthesis, and antioxidant capacity by improving soil conditions and enzyme activities; excess P suppresses these effects [93, 95]. Due to shared uptake pathways, high S forms Se–S complexes that limit Se accumulation [49, 96]. However, studies have also found that nano-Se exhibits a synergistic effect with S at low concentrations (0.5–5 μM), promoting the growth of crops and increasing the dry matter yield [66]. The combined application of silicon (Si) and Se has also been found to have significant synergistic effects, which can significantly improve the growth of crops [63, 80]. Microbial synergies (e.g. *Bacillus amyloliquefaciens*, phosphate-solubilizing bacteria, arbuscular mycorrhizal fungi) reshape rhizosphere microbiomes, mitigate Se stress, activate soil Se, and promote uptake [9, 16, 17, 40]. Selenizing bacteria (e.g. *Exiguobacterium* sp.) also enable efficient phytofortification [72]. These mechanisms provide a foundation for enhancing the Se fertilizer efficiency while minimizing ecological risks.

## Challenges and future prospects

Se acts as a double-edged sword for plants. The regulatory mechanisms of Se as affecting horticultural crops’ growth, stress resistance, and quality remain incompletely elucidated, with current research largely confined to laboratory settings, thus limiting the development of agricultural applications. Future research is suggested to include the following: (i) Mechanisms of Se uptake, transport, and metabolism. Deciphering molecular networks of Se absorption, translocation, and assimilation; identifying Se-specific transporters, regulatory genes, and their functions; and elucidating responses to excessive Se stress. (ii) Optimizing Se fertilization strategies. Screening optimal Se forms, dosages, combined fertilizer applications, and application methods; evaluating long-term impacts on soil microbiomes, groundwater, and ecosystems. (iii) Synergistic effects of Se on plant stress resistance. Investigating mechanisms of Se in biotic/abiotic stress tolerance using multiomics technologies; exploring synergies with phytohormones and endophytes to establish targeted regulation strategies. (iv) Enhancing Se supplementation efficiency and safety. The synergistic effects between bioactive compounds and Se in Se-enriched crops contribute to enhanced health benefits [36]. Consequently, moderate consumption of these crops in Se-deficient regions not only mitigates Se deficiency-associated diseases but also supports antioxidant and anticancer activities, while improving immune functions [6]. However, the processing-related Se losses are worth noting. For example, brewing conditions (time/temperature/water quality) affect organic Se dissolution in tea infusions [85], and Se-enriched broccoli loses >50% of its Se content during boiling [10]. The recommended dietary allowance (RDA) for Se is 55 μg/d [47], and the tolerable upper intake level (UL) for adults is 400 μg/d [10]. Although horticultural products primarily contain safe and efficient organic Se, moderate consumption remains essential to prevent Se toxicity.

## Data Availability

No data was used for the research described in the article.
